# Challenges in striving to simultaneously achieve multiple resource allocation goals: the pan-Canadian Oncology Drug Review (pCODR) example

**DOI:** 10.3402/jmahp.v4.31463

**Published:** 2016-07-21

**Authors:** Heather McDonald, Cathy Charles, Laurie Elit, Amiram Gafni

**Affiliations:** 1 Health Research Methodology (HRM) Program, McMaster University, Hamilton, ON, Canada; 2 Department of Clinical Epidemiology and Biostatistics, Centre for Health Economics and Policy Analysis, McMaster University, Hamilton, ON, Canada; 3 Clinical Epidemiology and Biostatistics, Obstetrics and Gynecology, McMaster University, Hamilton, ON, Canada

**Keywords:** resource allocation, health technology assessment, oncology, cancer, pan-Canadian oncology drug review (pCODR), drug reimbursement, drug funding, health policy

## Abstract

The pan-Canadian Oncology Drug Review (pCODR) makes recommendations to Canada's provinces and territories (except Quebec) to guide their cancer drug funding decisions. The objective of this paper is to explore, using an economic perspective and the pCODR as an example, the challenges associated with striving to simultaneously achieve the goals of maximizing health benefits with available resources and improving access to a more consistent standard of care across Canada. The first challenge concerns how to interpret the goals in order to determine how resources should be allocated to achieve each goal. The second challenge relates to whether, if pursued simultaneously, both goals can be achieved to the same extent that each goal could have been achieved alone with the same available resources. Regarding the first challenge, we illustrate that, due to a lack of definitional clarity, it is difficult to determine exactly how resources should be allocated in order to achieve the goal of improving access to a more consistent standard of care across Canada. Regarding the second challenge, we illustrate that choosing to strive for both of the pCODR goals simultaneously will likely be associated with tradeoffs in the extent to which one or both goals can be achieved (relative to what could have been achieved for each goal alone with the same available resources). We suggest that, if the pCODR and the provincial drug plan decision-makers it supports want to strive for both goals simultaneously, they must prioritize the goals and explicitly identify the tradeoffs associated with the prioritization. This will ensure that the consequences of striving to simultaneously achieve both goals are explicit, transparent, and predictable for provincial drug plan decision-makers, physicians, patients, caregivers, and society as a whole.

Healthcare systems face the complex challenge of determining how to provide healthcare to best achieve their goals within an environment of scarce resources. Resource scarcity (or affordability) means that, whatever resources are available, they are insufficient to support all possible activities. As a result of scarcity, healthcare systems are forced to make choices about how to allocate resources. They must decide what services to provide (and thus what not to provide), to whom, at what stage of a disease, and for how long, in order to best achieve their goals.

Decision-makers responsible for drug coverage plans (e.g. government agencies) face the same resource allocation challenge described above. In Canada, organizations such as the pan-Canadian Oncology Drug Review (pCODR) have been developed to make drug reimbursement recommendations to Canada's provincial and territorial Ministries of Health. Established in 2010 by Canada's provincial and territorial Ministries of Health as a separate body from Canada's Common Drug Review (CDR), the pCODR assesses the clinical evidence and cost-effectiveness of new cancer drugs and uses this information to make recommendations to the provinces and territories (except Quebec) to guide their drug funding decisions.^1^ The CDR provides recommendations on all other drugs in all other disease areas. Each province is ultimately responsible for funding, administering, and governing its own drug budget. Thus, after the pCODR recommends for a new cancer drug, each pCODR-participating drug plan then makes a decision on whether or not to accept the pCODR recommendation.

Because the pCODR is making recommendations to guide the allocation of publicly funded drug budget dollars, it is important that the pCODR's resource allocation goals or objectives (terms we use synonymously in this paper) are clearly understood by stakeholders (which include the pCODR-participating drug plans, physicians, patients, caregivers, and the general public). This will enable stakeholders to judge whether and to what extent these goals have been achieved. A commonly referred to, although not always explicitly stated, resource allocation goal for healthcare systems is to maximize health benefits (aggregated across a population) through the allocation of available resources. Although the pCODR does not explicitly state that this is its resource allocation goal, the pCODR uses cost-effectiveness analysis (CEA) to guide its reimbursement decision-making. The underlying premise of CEA is that the goal of society or decision-makers is to maximize the total aggregate health benefit conferred for a given level of resources (1–4). Therefore, the maximization of health benefits with available resources is an implied resource allocation goal of the pCODR. In addition to this implied goal, the pCODR stated that its objective was (when this study was conducted) ‘to build the foundation for a streamlined, national cancer drug review process that supports evidence-based decision-making’, which will ultimately ‘improve access to a more consistent standard of care across Canada, and bring clarity for patients, health professionals and industry about how, when and why drug funding decisions are made’ (5). Improving access to a more consistent standard of care across Canada is an equity (fairness) goal that will require resources to be allocated in a particular way in order to be achieved. Therefore, the pCODR appears to have two separate goals that, if they are to be achieved, will influence the allocation of scarce resources.

The objective of this paper is to explore, using an economic perspective and the pCODR as an example, the challenges associated with striving to simultaneously achieve the two resource allocation goals. In doing so, we first explain why an economic perspective is used (See: ‘Using an Economic Perspective to Explore the Challenges of Striving to Simultaneously Achieve the pCODR's Two Goals’). We then describe the nature of the challenges associated with striving to simultaneously achieve the two pCODR goals (See: ‘Challenges to Achieving the pCODR's Goals Simultaneously’). The challenges fall into two categories. The first challenge concerns how to interpret the goals in order to determine how resources should be allocated to achieve each goal. To address this challenge, we explore whether the goals are clearly and transparently defined and operationalized by the pCODR (See ‘Challenges to Achieving the pCODR's Goals Simultaneously - Definitional clarity of the goals’). The second challenge relates to whether, if pursued simultaneously, both goals can be achieved to the same extent that each goal could have been achieved alone with the same available resources. To address this challenge, we explore whether, with the same available resources, the drugs to which resources should be allocated in order to achieve the goal of maximizing health benefits with available resources alone are the same as the drugs to which resources should be allocated in order to improve access to a more consistent standard of care across Canada alone (See ‘Challenges to Achieving the pCODR's Goals Simultaneously - Striving to simultaneously achieve both pCODR goals’). The pCODR stated that its national cancer drug review process will bring clarity about how, when, and why drug funding decisions are made (5). Therefore, in the section entitled ‘Challenges to Achieving the pCODR's Goals Simultaneously - Does the pCODR transparently describe how it integrates its two goals?’, we discuss whether the pCODR transparently states how its two resource allocation goals are integrated in order to reach reimbursement recommendations. Finally, in the section entitled ‘Possible Tradeoffs Associated with Striving to Simultaneously Achieve the pCODR's two Goals’ we illustrate one of the consequences of the above challenges, namely, the tradeoffs in goal achievement that may be involved in striving to simultaneously maximize health benefits with available resources and improve access to a more consistent standard of care across Canada. Our use of the term ‘tradeoff’ refers to a reduction in the extent to which a particular goal can be achieved when multiple goals are simultaneously pursued, relative to when that single goal is pursued alone with the same available resources.

## Using an economic perspective to explore the challenges of striving to simultaneously achieve the pCODR's two goals

Economics is just one perspective that could be used to explore the challenges of striving to simultaneously achieve both of the pCODR's goals. However, we suggest that it is an appropriate perspective for several reasons. Economics is a discipline that studies how to allocate scarce resources in order to best achieve the goals stated by decision makers. Economics is based on the three fundamental concepts of scarcity (whatever resources are available, they are insufficient to support all possible activities), choices (because resources are scarce, we must choose between different ways of using them), and opportunity cost (by choosing to use resources in one particular way, we forego opportunities to use these same resources in any other way). These three fundamental concepts are relevant to the provincial drug budget resource allocation task that the pCODR recommendations are intended to guide. Due to resource scarcity (i.e. because there are a limited number of dollars available in the drug budgets), provinces must make choices regarding which drugs to publicly reimburse. Furthermore, these choices are associated with opportunity costs: by choosing to spend drug budget dollars on reimbursing certain drugs, the provinces forego the opportunity to use these drug budget dollars to reimburse any other drugs.

Scarcity, choices, and opportunity costs are also relevant to the challenge of striving to simultaneously achieve both of the pCODR's goals. If there was no resource scarcity, then Canada's provinces could simultaneously achieve both goals (unless contradictory) to the same extent that each could be achieved alone. However, resources are scarce. Consequently, if the type and mix of drugs that should be reimbursed in order to achieve each of the pCODR goals alone differ, then choosing to allocate resources in order to achieve one of the goals to its maximum extent with the available resources will have opportunity costs. These opportunity costs manifest as tradeoffs in the extent to which the other goal can be achieved.

## Challenges to achieving the pCODR's goals simultaneously

### Definitional clarity of the goals

To determine how resources should be allocated in order to best achieve a given goal, it is critical that the goal be clearly defined and operationalized. Below, we explore the pCODR's two goals to determine whether each is clearly defined and operationalized.

#### Goal 1: Maximizing Health Benefits With Available Resources

A clear definition of the goal of maximizing health benefits with available resources is not provided by the pCODR. Indeed, the goal itself is not explicitly stated and instead has been inferred by us based on the pCODR's use of CEA to guide reimbursement recommendations. The CEA literature, however, clearly states that the goal of CEA is to maximize the health of the population (1–4). Although the measure that should be used to quantify health gains under a CEA analysis is not explicitly defined in the literature, most people use quality-adjusted-life-years (QALYs). A QALY is a year of life that has been adjusted for its quality of life. With QALYs, an individual's health is measured as the product of a person's total years of life adjusted for quality, and a population's health is measured as the sum of QALYs for all individuals in the population. Though the way QALYs should be valued is also not explicitly defined in the literature, most people value them in the manner described by Wagstaff (6). That is, all QALYs are valued equally regardless of who gains or loses them.

Although the valuation of benefits for the health-benefit maximization goal is typically operationalized using QALYs in the manner we describe above, the goal could also be operationalized in other ways. For example, the assumption that all QALYs should be valued equally may not accurately capture important equity considerations related to the health benefits gained. Quality adjusted life-years could thus be valued in other ways, with health gains realized in certain disease areas or by certain populations being weighted more heavily than health gains in other areas. Of note, the National Institute for Health and Clinical Excellence (NICE) also explored this issue, reviewing the concept of QALY weighting as part of the 2011–2012 update to their guide for methods of technology appraisal (7). Although the goal of maximizing health benefits with available resources could be operationalized in different ways, we assume for the remainder of the paper that the operationalization is as described above, that is, via the use of QALYs in the manner described by Wagstaff (6).

#### Goal 2: Improving Access to a More Consistent Standard of Care Across Canada

The pCODR notes that its national, evidence-based drug review process will ultimately improve access to a more consistent standard of care across Canada (5). However, the phrases ‘access’, ‘more consistent’, and ‘standard of care’ are not defined, discussed, or operationalized any further. Consequently, numerous questions arise, some of which are outlined below.

First, what is the definition of ‘standard of care’, and by whom is the standard of care determined? Is the standard of care determined by what is recommended in clinical guidelines? For some types of cancer, there might be more than one clinical guideline describing how the disease should be managed once diagnosed, while for other types of cancer, there might not be any guidelines. If there are multiple guidelines, which guidelines should be referenced and based on what criteria is the guideline of choice selected? Alternatively, does the pCODR intend to determine the standard of care itself? Although the pCODR refers to itself as an evidence-based drug review process, its mandate does not include the development of clinical guidelines. Therefore, it is not clear how a pCODR-determined standard of care would align with other guidelines developed by the medical community. Furthermore, although the standard of care is often interpreted to mean the best possible care, this may not be possible in the case of the pCODR. Resources are scarce and the level of scarcity varies across provinces. Consequently, if the pCODR's goal is aimed at having the same cancer drugs reimbursed in all provinces, then the standard of care might be the least costly cancer drugs (which are often less effective or worse in some manner relative to more costly drugs for the same disease) that can be afforded by all provinces. As such, achieving the goal of ‘improving access to a more consistent standard of care across Canada’ may actually mean that the cancer drugs that get reimbursed across provinces are worse than currently available cancer drug options.

Second, what is meant by the phrase ‘more consistent’? To illustrate the challenges associated with interpreting this phrase, we assume that ‘standard of care’ means ‘the drugs recommended by the pCODR’, and that the pCODR goal is related only to the reimbursement (i.e. drugs are free of charge for beneficiaries at the point of consumption) of the drugs recommended as the standard of care on the provincial drug plans. Based on these assumptions, what has to happen in order for the standard of care to be considered ‘more consistent’? The word ‘more’ implies a level of gradation in terms of having consistency in the standard of care across Canada. Therefore, do only certain cancer drugs, but more than are currently reimbursed, need to be reimbursed across all provinces in order for the standard of care to be ‘more consistent’? If so, which drugs? Or, do all (or certain) cancer drugs need to be reimbursed in a certain number of provinces instead of in all provinces in order for the standard of care to be ‘more consistent’? Alternatively, instead of looking at the number of provinces in which cancer drugs are reimbursed, is the standard of care ‘more consistent’, if the total number of cancer patients in Canada for whom certain (or all) cancer drugs are publicly reimbursed increases?

Third, what is meant by the term ‘access’? Is the pCODR referring to the public reimbursement (i.e. the drug is free of charge at the point of care for drug plan beneficiaries) of cancer drugs, patients’ ability to physically access drugs, both of these, or something else? For a variety of reasons, there may be differences in patients’ ability to access a given drug even if it is publicly reimbursed across all provinces. Differences may include proximity to a medical center that can administer a given drug, or patient-related financial constraints such as the transportation and accommodation costs associated with treatment (if, e.g., a patient needs to reside near a cancer center over the course of the treatment). This has recently been observed by Chafe et al., who noted that, ‘While there is moderate consistency in the selection of cancer drugs that account for the highest provincial expenditures, considerable differences were found in the rates at which some drugs are accessed across provincial programs’ (8).

In summary, the phrases ‘standard of care’, ‘more consistent’, and ‘access’ can, as we have illustrated above, have multiple interpretations. This creates uncertainty for drug plan decision-makers because different interpretations of these phrases can have different implications in terms of how resources should be allocated in order to achieve the pCODR's equity goal of improving access to a more consistent standard of care across Canada. It also creates challenges related to the transparency and accountability of the pCODR's recommendations. From the perspective of transparency, the exact goal that the pCODR is trying to accomplish (as relates to consistency in standard of care) is unclear. From an accountability perspective, the multiple possible interpretations of the consistency goal make it difficult to determine whether the pCODR's recommendations and the provincial drug plan reimbursement decisions are consistent with the goal. Thus, although striving to improve access to a more consistent standard of care across Canada may seem like a benign and possibly beneficial goal, it is in reality a highly complex objective that requires further clarification before it can be incorporated into the resource allocation decision-making task in a meaningful way.

### Striving to simultaneously achieve both pCODR goals

Even if both pCODR goals were clearly defined and operationalized, we still need to determine whether they can be simultaneously achieved to the same extent that each single goal could have been achieved alone with the same available resources. To address this question, we first describe the resource allocation considerations associated with achieving each single goal alone.

#### i) Resource Allocation Considerations for Achieving a Single Goal of Maximizing Health Benefits with Available Resources

A goal of maximizing health benefits with available resources is based on striving to ensure that the available resources are spent on the activities that confer the maximum aggregate health benefits for the population. Typically, when considering whether to reimburse a new drug, drug plan decision-makers are working in an environment in which resources are already fully allocated (i.e. where the drug budget is already fully spent). Thus, in order to reimburse a new drug, resources have to be transferred away from activities that are currently receiving resources (i.e. currently being funded). Resources could come from elsewhere in the drug budget (by discontinuing or restricting the reimbursement of other drugs), from the overall healthcare budget (by discontinuing or restricting other healthcare activities), or from outside of healthcare (by discontinuing or restricting activities in other provincial ministries such as education or transportation). Reimbursing a new drug (for the typical case of a new drug that is more effective and more costly than what is currently reimbursed) will achieve the goal of maximizing health benefits with available resources if both of the following conditions are met:

1. There is an activity or combination of activities currently being funded that, if discontinued or restricted (in terms of funding), would free up enough resources to reimburse the new drug.
2. The total benefits that would be foregone by discontinuing or restricting the funding of these activities is less than the total health benefits that would be gained by reimbursing the new drug.^2^

Whether there is an activity or combination of activities from which resources can be transferred in order to reimburse a new drug and whether more health benefits will be gained than lost as a result depends on two factors: a population's medical needs and a decision-maker's total available resources. These factors, which are described below, vary across provinces. Consequently, whether a given new drug can be reimbursed in a way that maximizes health benefits with available resources is also likely to vary across provinces.

Medical Needs: The medical need for a new drug depends on a population's underlying demographics, socioeconomics, and epidemiology (9, 10). These factors, which vary across provinces, will drive the incidence and prevalence of the disease that the drug is intended to treat, which will in turn determine (1) the total amount of resources required to provide (i.e., reimburse) a new drug needed to treat that disease (and thus, the total amount of resources that need to be freed up) and (2) the total aggregate health benefits that will be gained by reimbursing that drug for those in need within the population. Variations across provinces in the incidence of different types of cancers can be found in the Canadian Cancer Society's 2015 Canadian Cancer Statistics report 2015 (i.e. Tables 2.4 and 2.5, cited in Ref. (11)).

A population's other medical needs (which are based on the prevalence of other diseases), if met, will determine the total resources presently allocated toward other healthcare activities (and thus, whether existing healthcare resources are already fully allocated), along with the total health benefits gained from those activities. A population's medical needs affect the total resources required to reimburse a new drug and the net gain (or loss) of aggregate health benefits that result from allocating resources away from currently funded activities (when necessary) and toward a new drug. Consequently, these medical needs must be factored in when trying to allocate resources in a way that will maximize health benefits.

Total Available Resources: A decision-maker's total available resources (i.e. the total available dollars) will determine which and how many activities can be funded. In the case of the provincial drug plan decision-makers, the total available resources (i.e. the total drug budget dollars) will determine which and how many drugs can be reimbursed. The total resources available to a drug plan decision-maker typically vary across provinces due to differences in the size of each provincial drug budget (e.g. the 2016–2017 public drug budgets for the provinces of Ontario, Quebec, and Alberta were approximately $5.0B, $3.8B, and $2.0B, respectively) (12–14) and the size and characteristics of the population served. Furthermore, whatever resources are available, they will be insufficient to pay for all possible activities, due to resource scarcity.

To illustrate how a decision-maker's total available resources determine whether reimbursing a new drug maximizes health benefits with available resources, we use an example based on two hypothetical provinces ‘Province A’ and ‘Province B’. Province A has a larger drug budget than Province B, but the two provinces are identical in all other variables (e.g. disease prevalence, size of population). Because of its larger drug budget, Province A can reimburse more drugs than province B. As a result, when a new and more costly drug is being considered for reimbursement it is more likely that, amongst the current drugs that are being reimbursed, one can find a candidate for dis-reimbursement (i.e., a drug or combination of drugs that, when dis-reimbursed, will free up enough resources to pay for the new drug and where the health benefits lost as a result of the dis-reimbursement are less than the health benefits gained from the new drug) in province A than in province B.

#### ii) Resource Allocation Considerations for Achieving a Single Goal of Improving Access to a More Consistent Standard of Care Across Canada

The different interpretations of the pCODR's goal of improving access to a more consistent standard of care across Canada can, as we highlighted in the section entitled ‘Challenges to Achieving the pCODR's Goals Simultaneously - Definitional clarity of the goals’, have different implications in terms how resources should be allocated. However, we make some simplifying assumptions in this section in order to provide some examples of how resources might have to be allocated in order to achieve this goal. In our first example, we assume that that the goal of improving access to a more consistent standard of care across Canada is aimed at having all of the pCODR-recommended drugs reimbursed in all provinces for provincial drug plan beneficiaries with the same medical need. We further assume that there are sufficient resources in all provinces to achieve this goal (i.e. resources that can be freed up by discontinuing currently funded activities). Based on these assumptions, and regardless of the impact that freeing up resources has on other currently funded activities, the consistency goal would be achieved if all provincial drug plans reimburse all of the pCODR-recommended drugs for drug plan beneficiaries with the same medical need.

The example above interprets the pCODR goal as an ‘all-or-none’ proposition (i.e. the only way to achieve the goal is for *all* provinces to reimburse *all* pCODR-recommended drugs for eligible beneficiaries). However, depending on how the word ‘more’ in the pCODR's goal is defined, there may be yet other ways for resources to be allocated in order to achieve the goal. This is particularly relevant in the event that there are insufficient resources to accomplish the all-or-none version of the goal that we used in our first example. To illustrate our point, we assume again that the goal is related to the reimbursement of pCODR-recommended drugs on the provincial drug plans. However, instead of assuming that all pCODR-recommended drugs have to be reimbursed in all provinces, we assume that the standard of care is considered to be ‘more consistent’, if each of the pCODR-recommended new drugs is reimbursed in at least three provinces for patients with the same medical need. In this case, only three provinces would have to allocate resources towards reimbursing each pCODR-recommended drug for eligible beneficiaries with the same medical need in order for the goal to be achieved. This is just one way that resources might have to be allocated in order to achieve the pCODR's consistency goal, depending on how ‘more consistent’ is defined and operationalized.

#### iii) Combining the Two pCODR Goals

The sections above describe how resources should be allocated in order to achieve the goal of maximizing health benefits with available resources alone or the goal of improving access to a more consistent standard of care alone. However, because both of these are pCODR goals, the question becomes whether the two goals can be simultaneously achieved to the same extent that each could have been achieved alone with the same available resources. To address this question, we again make some simplifying assumptions. First, we assume that the goal of improving access to a more consistent standard of care across Canada is aimed at having all of the pCODR-recommended drugs reimbursed in all provinces for provincial drug plan beneficiaries with the same medical need. We next assume, as an example, that the pCODR recommends Drug A to be reimbursed for patients with Cancer X. In this example, the pCODR's consistency goal will be achieved if all provinces reimburse Drug A for patients with Cancer X (assuming they have sufficient resources to do so and regardless of the impact this has on other currently funded activities). The health-benefit maximization goal, on the other hand, will be achieved if resources are allocated using the approach described in part i of ‘Challenges to Achieving the pCODR's Goals Simultaneously - Striving to simultaneously achieve both goals’. That is, reimbursing Drug A for patients with Cancer X will only maximize health benefits with available resources in a province if (1) there is an activity or combination of activities currently receiving resources that, if discontinued or restricted (funding-wise), would free up enough resources to reimburse the new drug and (2) the benefits that would be given up by discontinuing or restricting these activities are less than the health benefits that will be gained by reimbursing the new drug. It is possible that, in some provinces (say, e.g., in Province C and Province D), reimbursing Drug A for patients with Cancer X will maximize health benefits with available resources. If all of the provinces reimbursed Drug A for Cancer X for patients with the same medical need, then both of the pCODR goals will have been achieved to the same extent that each goal could have been achieved alone with the same available resources in Provinces C and D (as long as the drugs for which reimbursement must be discontinued in order to pay for Drug A are not other pCODR-recommended drugs or are the same pCODR-recommended drugs). However, because of differences in medical needs and total available resources, the mix and type of drugs that will maximize health benefits with available resources is expected to vary across provinces. Therefore, although health benefits may be maximized in Provinces C and D by reimbursing Drug A for patients with Cancer X, reimbursing Drug A for patients with Cancer X in the other provinces may lead to a loss of total health benefits. In the latter provinces, the health-benefit maximization goal will not have been achieved to the same extent that it could have been achieved alone with the same available resources. Furthermore, due again to different medical needs and total available resources, while Provinces C and D may be able to achieve both pCODR goals to the same extent that each goal could have been achieved alone by reimbursing Drug A for Cancer X, reimbursing other pCODR-recommended drugs may not result in a net gain of aggregate health benefits in these same provinces. Therefore, for any given pCODR-recommended drug, it will likely not be possible to simultaneously achieve both of the pCODR goals across all provinces to the same extent that each goal could have been achieved alone with the same available resources.

### Does the pCODR transparently describe how it integrates its two goals?

If the two pCODR goals cannot be simultaneously achieved to the same extent that each could have been achieved alone with the same available resources (a likely scenario, as we suggest above), then the goals will need to be prioritized. In other words, decisions have to be made regarding which goal is more important to achieve. At this point, it is not clear how the pCODR integrates the two goals in order to make reimbursement recommendations because there are no explicit, public statements from the pCODR regarding the relative importance of each goal.

## Possible tradeoffs associated with striving to simultaneously achieve the pCODR's two goals

Because it will not always (and we think, in most cases) be possible to simultaneously achieve both of the pCODR goals to the same extent that each could have been achieved alone with the same available resources, tradeoffs will be incurred. The nature and magnitude of the tradeoffs will vary depending on (1) how each goal is defined and operationalized and (2) how the goals are prioritized. Due to the lack of definitional clarity regarding the consistency goal and because the pCODR has not indicated any prioritization of the goals, the exact tradeoffs associated with striving to simultaneously achieve the two pCODR goals are difficult to fully delineate. However, it is still important to understand what some of these tradeoffs could be. In order to illustrate some of the possible tradeoffs we make the simplifying, hypothetical assumptions outlined below.

1. The pCODR's consistency goal is prioritized over the goal of maximizing health benefits with available resources and is treated as a constraint (i.e. resources must be allocated to achieve the consistency goal in full as we define it in assumption 2 below), assuming that there are sufficient resources to do so and regardless of where the resources come from to satisfy this constraint.
2. The pCODR's goal of improving access to a more consistent standard of care across Canada is aimed at having all of the pCODR-recommended cancer drugs publicly reimbursed (i.e. the drug is free of charge at the point of consumption) in all provinces for all provincial drug plan beneficiaries with the same medical need.
3. The provincial drug budgets are fixed and fully allocated, and the resources required to reimburse a new drug will have to come from these budgets (i.e. they will not be taken from other budgets within the Ministries of Health or from other ministries). Note: In some provinces, there are separate budgets for cancer versus non-cancer drugs. We assume that, even in provinces with a separate cancer drug budget, the resources required to reimburse a new drug have to come from within the combined budgets for cancer and non-cancer drugs.

We acknowledge that the assumptions above may not always be applicable in reality. However, we make them to illustrate some of the tradeoffs associated with trying to simultaneously achieve the two pCODR goals. Even with these simplifying assumptions, the factors that must be taken into account in order to determine the tradeoffs are highly complex. More realistic situations may stray from these basic assumptions, such that determining the tradeoffs becomes even more complex.

For our illustration, we assume again that the pCODR recommends Drug A be reimbursed for patients with Cancer X. Based on the assumptions above, all provinces will have to reimburse Drug A for patients with Cancer X in order to achieve the consistency goal. To do this, resources will have to be transferred away from currently reimbursed drugs. However, as described in part iii of ‘Challenges to Achieving the pCODR's Goals Simultaneously - Striving to simultaneously achieve both goals’, discontinuing or restricting the reimbursement of currently funded drugs in order to reimburse Drug A may result in more health benefits being lost than are gained in at least some provinces. In these instances, the health-benefit maximization goal cannot be simultaneously achieved alongside the consistency goal to the same extent that it could have been achieved alone with the same available resources. Furthermore, the specific drugs for which reimbursement is discontinued or restricted, and the associated total health benefits that have to be foregone in order to reimburse Drug A, are likely to vary across provinces due to differences in medical needs and total available resources. Therefore, *the degree to which* the health-benefit maximization goal is impeded will also be different across provinces. Finally, depending on what is currently reimbursed in a province and on how many resources need to be freed up, the resources required to reimburse Drug A may have to come from discontinuing or restricting the reimbursement of cancer drugs that are presently reimbursed in the provinces (e.g. cancer drugs that were reimbursed in the provinces before the establishment of the pCODR). Thus, unless discontinuing the reimbursement of currently funded cancer drugs is specifically prohibited in the definition and operationalization of the pCODR goals, achieving the pCODR's consistency goal may have the unintended consequence of creating new inequalities across Canada in the public reimbursement of currently funded cancer drugs.

In making the assumption for illustrative purposes that the pCODR's consistency goal is a constraint, we demonstrate in the above scenarios that the health-benefit maximization goal is achieved to a lesser extent than it could have been achieved alone with the same available resources. This is one example of how the pCODR goals can be integrated, but there are also other ways of integrating the goals. For example, each goal could have a weighting in terms of relative importance instead of one goal fully trumping the other. This type of integration would result in different types of tradeoffs relative to what we have described in our example above (e.g. instead of only one goal being achieved to a lesser extent, both goals might be achieved to a lesser extent than each goal could have been achieved alone with the same available resources). In order to determine, in these cases, the exact degree to which each goal is traded off against achievement of the other goal, the weighting of each goal would have to be transparently described by the pCODR.

## Discussion

This paper highlights the challenges associated with striving to simultaneously achieve more than one resource allocation goal and uses, as an example, the pCODR's goals of maximizing health benefits with available resources and improving access to a more consistent standard of care across Canada. First, due to a lack of definitional clarity, it is difficult to determine exactly how resources should be allocated in order to achieve the pCODR's goal of improving access to a more consistent standard of care across Canada. Second, due to different medical needs across provinces and the total available resources per beneficiary, it is likely that the mix and type of drugs that should be reimbursed in order to maximize health benefits with available resources will vary across provinces, whereas the same cancer drugs would have to be reimbursed across all provinces in order to achieve the goal of improving access to a more consistent standard of care across Canada (if this latter goal is defined as we suggested in the section entitled ‘Possible Tradeoffs Associated with Striving to Simultaneously Achieve the pCODR's two Goals’). Consequently, choosing to strive for both of these goals simultaneously will likely be associated with tradeoffs in at least some provinces in the extent to which one or both of the goals can be achieved (relative to what could have been achieved for each goal alone with the same available resources.

Striving to simultaneously achieve the pCODR's two resource allocation goals and incurring tradeoffs in the extent to which the goals can be achieved might not necessarily be considered a bad thing. It is possible that these tradeoffs are acceptable to the pCODR and provincial drug plan decision-makers. The pCODR and the provincial drug plan decision-makers may decide, for example, that improving access to a more consistent standard of care across Canada is an important goal and some reduction in the ability to maximize health benefits with available resources is acceptable in order to achieve this consistency (or vice versa). However, there are no explicit statements from the pCODR about such tradeoffs, and thus it is not clear that the pCODR (or in turn the provincial drug plan decision-makers) are presently aware that tradeoffs will likely be incurred in striving to simultaneously achieve these two goals. If the pCODR is aware of these tradeoffs, we suggest that it is incumbent on them to clearly and transparently specify the nature and magnitude of the tradeoffs so that they are transparent for drug plan decision-makers, physicians, patients, caregivers, and the general public.

There is yet another challenge with striving to simultaneously achieve both of the pCODR goals. Because achievement of the consistency goal, if defined as we suggested in the section entitled ‘Possible Tradeoffs Associated with Striving to Simultaneously Achieve the pCODR's two Goals’, must be effected nationally, then all of the pCODR-participating provinces would have to agree to (1) the existence of and (2) the definition of the consistency goal. Furthermore, they would all have to accept the tradeoffs associated with striving to simultaneously achieve both of the pCODR goals, even though the magnitude and nature of the tradeoffs are likely to vary across provinces (in addition to varying depending on the cancer drug under consideration). Presently, it is not clear that all pCODR-participating provinces have agreed to a consistency goal that requires all provinces to reimburse the same cancer drugs or to the tradeoffs associated with striving to simultaneously achieve both pCODR goals. Underscoring this point is the fact that some provinces maintain their own cancer advisory bodies to provide province-specific cancer drug reimbursement advice. For example, Ontario has recently established the Ontario Steering Committee for Cancer Drug Programs. Noting that ‘there remains a need to obtain arms-length cancer-specific policy and program advice to support Ontario's cancer drug reimbursement programs and processes’, this steering committee will, among other things, provide guidance on Ontario-specific cancer drug funding policies and decisions (15). Finally, there is a fundamental paradox associated with striving for consistency across Canada in the public reimbursement of cancer drugs given that Quebec, which represents approximately 24% of the Canadian population, does not participate in the pCODR process (16).

A potential criticism of our arguments may be that we have misinterpreted the goal of improving access to a more consistent standard of care across Canada (5). However, we suggest that our interpretation (i.e. the goal is aimed at having the same cancer drugs reimbursed across Canadian provinces) is not outside the realm of what might be intended by the pCODR (or outside the realm of how drug plan decision-makers might interpret this goal) for two reasons. First, the pCODR is responsible for making recommendations regarding which drugs should be reimbursed on the provincial drug plans (which supports our illustrative assumption that the goal is related to drugs and not other treatment modalities). Second, variations across the provinces in cancer drug reimbursement was cited as a reason for the establishment of the JODR (which ultimately evolved into the pCODR), supporting our illustrative assumption that the pCODR's goal is aimed at having the same drugs reimbursed across all provinces (17). Our assumptions notwithstanding, if there is another intended definition and operationalization of this goal, it is incumbent upon the pCODR to state this clearly and transparently. Arguments that we have misinterpreted the goal simply serve to underscore the challenges we have highlighted in this paper about how to interpret the goal in order to allocate resources in a way that best achieves the goal.

Another possible criticism of the arguments we have laid out in this paper may be that we are incorrect in inferring that the pCODR has an underlying goal of maximizing health benefits with available resources. We suggest that, even without an explicit statement of this goal, maximizing health benefits with available resources is an implied goal that underpins the pCODR's work. This is because the pCODR uses CEA as a key component of its reimbursement review process and, as noted in the CEA methodology literature, the underlying premise of CEA is that the goal of society or society's decision-makers is to maximize the total aggregate health benefit conferred to the population for a given level of resources (1–4).

Although we use the pCODR as a reference point in this paper, the pCODR is not the first or only agency to have a goal related to having a more consistent standard of care nationally alongside a goal of maximizing health benefits with available resources. For example, when NICE first started conducting assessments (called ‘technology appraisals’) to guide the National Health Service (NHS) and local health authorities on which medicines and treatments should be publicly reimbursed its goals included ‘… maximizing the health gain from the use of NHS resources …’ and ‘… to remove unfairness in the availability of technologies in different localities and to minimize the possibility of further examples of unfairness or inequity being introduced’ (18). The NHS also notes that ‘NICE's technology appraisals program is designed to ensure that people across England and Wales have equal access to new and existing medicines that are deemed clinically and cost effective, reducing the risk of a postcode lottery of care’ (19). Therefore, the challenges we highlight in this paper are relevant not only for the pCODR but also for other drug reimbursement review agencies (and their respective stakeholders) that are (or are considering) striving to achieve both of these goals simultaneously. There may also be healthcare initiatives outside the realm of drug reimbursement in which a goal of achieving national consistency is sought. Given that maximizing health benefits with available resources is a frequently cited goal for healthcare systems, the challenges of striving to simultaneously achieve both goals would similarly apply. Finally, these challenges would be even more complex in scenarios in which decision-makers try to simultaneously achieve more than two goals that, if they are to be achieved, influence the allocation of scarce resources.

## Conclusion

In this paper, we highlight some of the challenges associated with striving to simultaneously achieve the pCODR's goals of maximizing health benefits with available resources and improving access to a more consistent standard of care across Canada. In addition to the challenges related to the definitional clarity of the consistency goal, we suggest that it will likely not be possible to simultaneously achieve both goals across all provinces to the same extent that each goal can be achieved alone with the same available resources. Therefore, if the pCODR and the provincial drug plan decision-makers it supports want to strive for both of these goals simultaneously, they need to prioritize the goals and explicitly identify the tradeoffs associated with the prioritization. This will ensure that the consequences of striving to simultaneously achieve both goals are made explicit, transparent, and predictable for provincial drug plan decision-makers, physicians, patients, caregivers, and society as a whole.

## Footnotes

1 From this point forward, we refer to provinces and territories as ‘provinces’ for simplicity.

2 Even if the drug budget can be increased in order to reimburse new drugs (such that resources do not have to be transferred away from other activities), the (cancer) drugs will still be competing for more resources than there are resources available because of resource scarcity. Therefore, if the goal is to maximize health benefits with available resources, then decision-makers must still consider what is foregone by using the available resources to reimburse a new drug instead of using the same resources in any other way.
